# Supplementary material for: HLA-B*27:05 alters immunodominance hierarchy of universal influenza-specific CD8+ T cells
Source: PLoS Pathog. 2020 Aug 4;16(8):e1008714. doi: 10.1371/journal.ppat.1008714 (PMC7428290; doi:10.1371/journal.ppat.1008714)
Supplement: Attachment — Submitted filename: Response_to_reviewers PLoS Path.docx [file ppat.1008714.s002.docx]

**RESPONSES TO REVIEWERS’ COMMENTS**

We thank the Reviewers for their highly favourable comments. Specific responses to the Reviewers’ comments are outlined in a point-by point form.

**REVIEWER 1 (Comments to the Author):**

*In this work, the authors studied the immunodominance status of influenza-specific universal CD8+ T-cells in HLA-I heterozygote individuals, which express two or more universal HLAs for Influenza A. The article brings interesting data showing that, while CD8+ T-cell responses directed towards A2/M158 are generally immunodominant, this response is markedly diminished in individuals expressing also the HLA-B*27:05. The authors show that A2+B27- individuals present highly functional TRBV19/TRAV27 TCRab clonotypes, but that in B27+ persons, A2 cells present increased diversity and increased prevalence of private less functional clonotypes, explaining partially the differential immunodominance of responses. The immunodominance of B27 restricted T-cells has been already demonstrated over the B8 ones (Boon et al., 2002), and, in experimental mice models, the co-expression of B7 and B27 alleles is associated with a B7 immunodominant responses (Akram and Inman, 2013). Although the immunodominance in the context of influenza infection has been already demonstrated in several studies, this article is interesting in showing that HLA-B27 alters the immunodominance hierarchy and functional properties of universal influenza-specific CD8+ T cells, impacting the response of the most prevalent HLA across multiple ethnicities. The understanding of immunodominance in the context of Influenza infection is crucial for the development of T-cells based therapy. The manuscript is clear and well written. The present group has a recognize expertise and many high-quality publications in the field of CD8+ T cell responses in Influenza infection. I have only minor comments.*

We thank the Reviewer for these favourable comments.

*Minor issues:*

*• In keywords: correct the word “immunodominance”;*

We have corrected the word “immunodominance” in keywords online

*• In abstract, replace the full stop after “capacity” with a comma;*

We have made this modification in the Abstract

*• In results, correct the text for Figure 2C (line 204). It’s indicating Figure 5C;*

We have replaced Figure 5C with Figure 2C in line 204

*• About the data Figure 6 and related text , is it correct to conclude that B27 cells are more proliferative than A2 cells based on the numbers of tetramer+ cells and IFN-gamma production upon stimulation?*

We agree. B27-NP CD8^+^ T cells are more proliferative than A2-M1 CD8^+^ T cells.

*• Can the authors discuss the fact that A2 and B27 cells (i) present different phenotypes, (ii) althodo not differ in terms of cytotoxins expression?*

Following the Reviewer’s comment, we have added the following sentence to our Discussion (page 9, line 383):

However, while HLA-A*02:01^+^M1_58_^+^ and HLA-B*27:05^+^NP_383_^+^ CD8^+^ T cells are of different phenotypes, they do not differ in terms of CD107a expression, a marker of T cell degranulation and thus cytotoxicity.

**REVIEWER 2 (Comments to the Author):**

*This is a very well-written manuscript investigating CD8 T cell immunodominance in HLA heterozygotes. The authors have found that A2/M1 CD8 T-cells in non-HLA-B*27:05 individuals were immunodominant, contained public TCR clonotypes and displayed highly polyfunctional and proliferative capacity. However, in HLA-B*27:05-expressing donors, A2/M1CD8 T-cells were subdominant, with distinct TCR clonotypes and diminished avidity, proliferation and polyfunctionality. This study will not only help us understand the protective CD8 T cell responses following influenza infection in different individuals, but also have significant impact on the future design of CD8 T cell-based influenza vaccines. The reviewer only has one minor comment regarding the manuscript: if the information is available to the authors, please list age, sex and ethnicity information of the blood donors in a table during revision.*

We thank the Reviewer for these comments.

*Minor issues:*

*If the information is available to the authors, please list age, sex and ethnicity information of the blood donors in a table during revision.*

Table 1 lists age of the donors. Gender and ethnicity were not available for the majority of the donors.
